# Correction to: Multidisciplinary recommendations for essential baseline functional and laboratory tests to facilitate early diagnosis and management of immune‑related adverse events among cancer patients

**DOI:** 10.1007/s00262-023-03456-w

**Published:** 2023-05-08

**Authors:** Berna C. Özdemir, Cristina Espinosa da Silva, Dimitri Arangalage, Pierre Monney, Sabina A. Guler, Uyen Huynh-Do, Guido Stirnimann, Lucia Possamai, Roman Trepp, Robert Hoepner, Anke Salmen, Camille L. Gerard, Petr Hruz, Lisa Christ, Sacha I. Rothschild

**Affiliations:** 1 Department of Medical Oncology, Inselspital, Bern University Hospital, University of Bern, Bern, Switzerland; 2 Herbert Wertheim School of Public Health and Human Longevity Science, University of California, San Diego, La Jolla, CA USA; 3 Division of Epidemiology and Biostatistics, School of Public Health, San Diego State University, San Diego, USA; 4 Department of Cardiology, INSERM U1148, Bichat Hospital, University of Paris, Paris, France; 5 Department of Cardiology, Lausanne University Hospital (CHUV), University of Lausanne, Lausanne, Switzerland; 6 Department of Pulmonary Medicine, Inselspital, Bern University Hospital, University of Bern, Bern, Switzerland; 7 Department of Nephrology and Hypertension, Inselspital, Bern University Hospital, University of Bern, Bern, Switzerland; 8 Department of Visceral Surgery and Medicine, Inselspital, Bern University Hospital, University of Bern, Bern, Switzerland; 9 Department of Metabolism, Digestion and Reproduction, Faculty of Medicine, Imperial College London, London, UK; 10 Department of Diabetes, Endocrinology, Nutritional Medicine and Metabolism (UDEM), Inselspital, Bern University Hospital, University of Bern, Bern, Switzerland; 11 Department of Neurology, Inselspital, Bern University Hospital, University of Bern, Bern, Switzerland; 12 Department of Oncology, Lausanne University Hospital (CHUV), University of Lausanne, Lausanne, Switzerland; 13 The Francis Crick Institute, London, UK; 14 Department of Gastroenterology, University Hospital Basel, Basel, Switzerland; 15 Department of Rheumatology and Immunology, Inselspital, Bern University Hospital, University of Bern, Bern, Switzerland; 16 Department of Medical Oncology, University Hospital Basel, Basel, Switzerland; 17 Department Internal Medicine, Center for Oncology and Hematology, Cantonal Hospital Baden, Baden, Switzerland

**Correction to: Cancer Immunology, Immunotherapy** 10.1007/s00262-023-03436-0

The original version of this article unfortunately contained a mistake. In the Table 1, some of the endocrine tests every 3–6 months are misplaced under the column "every 4–6 weeks".

The correct Table 1 should be as indicated in the next page.

**Table 1:** Essential laboratory and functional tests to evaluate before and during treatment with immune checkpoint inhibitors (ICIs)

	Prior to ICI treatment	During ICI treatment			
	Baseline	During the first 3 months		After the first 3 months	
		Every ICI administration	Every 4–6 weeks	Every 4–6 weeks	Every 3–6 months
Liver	AST, ALT, GGT, ALP, total bilirubin	AST, ALT, GGT, ALP, total bilirubin	–	AST, ALT, GGT, ALP, total bilirubin	–
Endocrine function	Cortisol, TSH, fT3, fT4, AMH ^a^, FSH, LH, Estradiol ^b^, Glucose, Hb1Ac	TSH, fT4, fT3	–	–	TSH, fT4, fT3, AMH, FSH, LH, Estradiol, Hb1Ac
Heart	ECG, CK, Troponin T and I Echocardiography^c^	CK, Troponin T and I^d^	–	CK, Troponin T^d^	–
Kidney	Creatinine, Urea, Urine dipstick	Creatinine, Urea	–	Creatinine, Urea	Urine dipstick
Fluid and electrolyte balance	Sodium, Potassium	Sodium, Potassium	–	Sodium, Potassium	–
Bone metabolism	Calcium, Albumin, Vitamin D_3_	–	–	–	Calcium, Albumin
Bone marrow	Complete blood count	–	Complete blood count	Complete blood count	–
Latent infections	Hepatitis B and C, HIV, Tuberculosis	–	–	–	–
Coagulation and fibrinolysis	INR, PT, PTT, D-Dimer, Fibrinogen, Ferritin	–	–	–	INR, PT, PTT
Gastrointestinal function	FC, C.diff, CMV^e^	–	–	–	–
Lung function	Spirometry and DLCO^f^	–	–	–	–
Neurological function	Neurological examination (cranial nerves and motoric and sensory function)	–	–	–	–

*ALP* alkaline phosphatase, *ALT* alanine aminotransferase, *AST* aspartate aminotransferase, *CK* creatine kinase, *DLCO* diffusing capacity for carbon monoxide, *ECG* electrocardiogram, *FC* fecal calprotectin, *FSH* follicle-stimulating hormone, *GGT* gamma-glutamyl transpeptidase, *INR* international normalized ratio, *LDH* lactate dehydrogenase, *LH* luteinizing hormone, *PT* prothrombin time, *PTT* partial thromboplastin time, *TSH* thyroid-stimulating hormone

^a^Female patients 18–42 years old

^b^Premenopausal female patients

^c^Patients at risk of developing ICI-related myocarditis (e.g., combination ICI, previous cardiotoxic therapies, cardiovascular diseases)

^d^During the first 4 doses before every ICI administration, thereafter every 3 doses

^e^Patients with preexisting diarrhea

^f^Patients with preexisting lung disease or radiotherapy to the lung or mediastinum

